# Editorial: Colorectal cancer awareness month 2023: diagnosis, clinical course, and surgical management of metastatic colorectal cancer

**DOI:** 10.3389/fonc.2024.1496480

**Published:** 2024-10-14

**Authors:** Aldo Rocca, Alfonso Reginelli, Luca Viganò

**Affiliations:** ^1^ Department of Medicine and Health Science “V. Tiberio”, University of Molise, Campobasso, Italy; ^2^ Hepatobiliary and Pancreatic Surgery Unit, Pineta Grande Hospital, Castel Volturno, Caserta, Italy; ^3^ Department of Precision Medicine, University of Campania “L. Vanvitelli”, Naples, Italy; ^4^ Hepatobiliary Unit, Department of Minimally Invasive General and Oncologic Surgery, Humanitas Gavazzeni University Hospital, Bergamo, Italy; ^5^ Department of Biomedical Sciences, Humanitas University, Milan, Italy

**Keywords:** radiomic, precision surgery, colorectal cancer, prevention and diagnosis, treatment strategy

Despite advances in medical care, Colorectal Cancer (CRC) is still a challenging health problem, representing the third most common cancer in terms of incidence and one of the leading causes of cancer-related mortality in the Western world (1).

Researchers are addressing various aspects of CRC, encompassing prevention, diagnosis, and treatment at different stages, from precancerous lesions to locally advanced and metastatic tumors (2–6). After pandemic era (7), advances in technology and cancer research are rapidly providing valuable and innovative insights for clinicians, researchers, and policymakers. Physicians must make clinical decisions among several options, pursuing a personalized approach for each patient in a continuously evolving scenario. Multidisciplinarity is the key, combining the expertise of medical oncologists, surgical oncologists, radiation oncologists, gastroenterologists, endoscopists, interventional radiologists, nuclear medicine physicians, pathologists, palliatives, and nurses in a patient-centered process (8–12). It is the only way to provide cutting-edge treatments, balancing benefits, risks, and costs in light of the latest evidence.

Following the considerations above, this editorial introduces a collection of articles entitled: “Colorectal Cancer Awareness Month 2023: Diagnosis, Clinical Course, and Surgical Management of Metastatic Colorectal Cancer,” which deeply explores the actual complexity of research and clinical practice for CRC patients.

In such a heterogeneous scenario, we have merged preclinical and clinical research on CRC, aiming to outline a single fil-rouge. Progress is driven by hyper-specialized research activity, but interconnection among different specialists is crucial for translating discoveries into clinical practice. Considering the latest innovations in each field, a multidisciplinary approach is essential to integrate them into the best clinical solution. For instance, medical and surgical oncologists strongly demand new biomarkers to guide their daily clinical decisions reliably (1, 13–15). The answer will likely be found by combining the results of genetic, radiomic, pathologic, and preclinical studies ongoing worldwide (16, 17). This is the spirit of the present Research Topic, in which the reader will navigate through the most recent advances and challenges in CRC research and treatment, always perceiving a unique aim: to build a multidisciplinary benchmark to answer clinical questions. Here are some of the most relevant insights from the published articles.

Schnitzer et al. analyzed the cost-effectiveness analysis of different imaging modalities for detecting colorectal liver metastases eligible for hepatic resection.

This study is critical for optimizing clinical outcomes and resource allocation, underscoring the importance of economic considerations in modern clinical reasoning (18–20).

At the same time, as reported by Zhang et al., the most recent laboratory researches, ranging from circulating biomarkers to single-cell RNA sequencing and bulk RNA transcriptome sequencing, pave the way to new approach to CRC, by unveiling heterogeneous immune landscape and identifying pivotal cell subpopulations associated with prognosis.

New biomarkers for CRC were also discussed. Lucarelli et al. proposed a potential role for the superior mesenteric vein ectasia in predicting liver metastases from rectal cancer, while Wang et al. suggested that serum mannose levels could predict the tumor N staging. A deeper knowledge of tumor biology is the basis for new therapeutic targets, which are crucial for developing personalized medicine approaches, and better prognostic models (21).

The term “Personalized medicine”, beside its therapeutic implication, also underscores the need for a patient-centered care. The patients and their relatives should be actively involved in the decision-making process and their feelings and behavior (o life-style) should be considered. According to the study by Gu et al., this remains an unmet need, with several patients not having a dominant role in clinical decision-making.

This issue is even more relevant in elderly patients: as reported by Huemer et al., patients aged 70 years or older with metastatic CRC can be effectively treated, but their comorbidities and specific physiological responses to treatment must be considered to find the right balance between efficacy and tolerability of interventions (22, 23). Ding et al. reported in a propensity score matching study the benefit of regional block techniques on postoperative high-grade complications in elderly patients with thoracic and abdominal cancers.

On the same line, the meta-analysis by Zeng et al. outlined the risk factors for lateral pelvic lymph node metastases in patients with low rectal cancer. The non-invasive stratification of risk for nodal metastases is essential to plan treatment and modulate aggressiveness of surgery. Finally, the increasing minimal invasiveness of therapeutic options enhances the access to treatment.

Shi et al., using a large database of over 25,000 patients, elucidated the indications for endoscopic treatment of T1N0 tumors, which offers a chance of cure even for frail patients.

In conclusion, this Research Topic aims to provide a deeper understanding of CRC, offering readers new perspectives on diagnosis, treatment, and patient management. The proposed papers strongly highlight the commitment of the medical and scientific community to improving the lives of patients affected by CRC through rigorous research and innovative solutions. While new complexities are continuously uncovered, the collected manuscripts provide invaluable contributions to shaping the future of CRC treatment.

In conclusion, this Research Topic aims to provide a deeper understanding of CRC, giving readers new perspectives concerning diagnosis, treatment, and patient management. From the proposed papers, it emerges with strength the commitment of the medical and scientific community to improve the lives of patients affected by CRC through rigorous research and innovative solutions. While new complexities are continuously uncovered, the collected manuscript give an invaluable contribution in shaping the near future of CRC treatment. As we continue to uncover the complexities of this disease, such contributions are invaluable in shaping the future of colorectal cancer care.
